# Barriers and facilitators to the HPV vaccine: a multicenter qualitative study of French general practitioners

**DOI:** 10.1186/s13690-023-01227-8

**Published:** 2024-01-04

**Authors:** Arthur Tron, Vincent Schlegel, Juliette Pinot, Sébastien BRUEL, Marie Ecollan, Josselin Le Bel, Louise Rossignol, Aurélie Gauchet, Amandine Gagneux-Brunon, Judith Mueller, Anne-Sophie Banaszuk, Nathalie Thilly, Serge Gilberg, Henri Partouche

**Affiliations:** 1 Faculté de Santé, Département de médecine générale, Université de Paris, UFR de Médecine – Site Cochin, 24, rue du Faubourg Saint-Jacques, Paris, F-75014 France; 2 Institut de recherche et de documentation en économie de la santé (IRDES), 117 bis rue Manin, Paris, 75019 France; 3 Department of General Practice, Jacques Lisfranc Faculty of Medicine, Saint-Etienne-Lyon University, Saint-Etienne, France; 4 Univ Lyon, Université Claude Bernard Lyon 1, Lyon, P2S UR4129, F-69008 France; 5 CIC-INSERM 1408, Saint-Etienne University Hospital, Saint-Etienne, France; 6 Laboratory of Psychology, University Grenoble Alps, Grenoble, France; 7 Centre International de Recherche en Infectiologie, Team GIMAP, Univ Lyon, Université Jean Monnet, Université Claude Bernard Lyon 1, Inserm, U1111, CNRS, UMR530, Lyon, France; 8 EHESP French School of Public Health, Institut Pasteur, Paris cedex 15, Paris, France; 9 Centre régional de Coordination des Dépistages des Cancers-Pays de la Loire, 5 rue des Basses Fouassières, Angers, 49000 France; 10 Université de Lorraine, APEMAC, Nancy, F-54000 France; 11 Département Méthodologie, Promotion, Investigation, Université de Lorraine, CHRU-Nancy, Nancy, F-54000 France

**Keywords:** Vaccine hesitancy, Mass vaccination, Vaccination refusal, Papillomavirus infections, Primary prevention, General practice, Primary care

## Abstract

**Background:**

In France, human papillomavirus (HPV) vaccination coverage is low, with 30.7% of 17-year-old girls having received a complete HPV vaccination schedule in 2020.

**Aim:**

To determine the perspective and behaviors of general practitioners (GPs) regarding HPV vaccination with their patients and if a reluctance is observed.

**Design and setting:**

A qualitative study based on semi-directed individual interviews was conducted between December 2019 and December 2020. A representative sample of GPs with various profiles were included in 4 French regions.

**Method:**

A purposive sampling was used and interviews were continued until data saturation was reached. The analysis was based on the grounded theory.

**Results:**

Twenty-six GPs aged 29–66 years were interviewed. The measures taken by the French health authorities (lowering the target age, reimbursing the vaccine, extending the target population to boys) were perceived as facilitators. The reported barriers were organizational, due to low attendance of adolescents, and relational, mainly due to parental vaccine hesitancy. Physicians had to deal with fears about the perceived risks and concerns about sexuality conveyed by HPV vaccination and linked to the socio-cultural characteristics of the families. Physicians developed strategies, including scientific knowledge mobilization, empowerment of families by promoting health through prevention, repetition of the vaccination proposals, personal experience and relationship. Different practices were identified according to three GP typologies: effective, convinced but unpersuasive, and reluctant physicians.

**Conclusion:**

Based on these results, specific interventions, including communication techniques, especially for hesitant or unpersuasive physicians, are needed to enable GPs to become more effective.

**Supplementary Information:**

The online version contains supplementary material available at 10.1186/s13690-023-01227-8.

**Table:** 

Text box 1. Contributions to the literature
• HPV vaccination coverage in the target population remains low in France.
• Most GPs develop strategies to deal with families' fear of vaccines and concerns about sexuality.
• Three GP typologies were found: effective, convinced but unpersuasive, and reluctant physicians.
• A part of French GPs remains hesitant about HPV vaccination.
• Communication training and a decision support tool could help physicians offer the HPV vaccine.

## Introduction

While vaccines play a crucial role in preventing infectious diseases, their use in the general population is challenged by doubts about their effectiveness and growing concerns about their potential adverse effects (AEs) [1]. France is not spared by this phenomenon: while in 2000, only 8% of French people reported being hesitant about vaccines, this figure rose to 41% in 2016 [2]. In France, “vaccine hesitancy”, that refers to delay in acceptance or refusal of vaccination despite availability of vaccination services, [1, 3, 4] relates more specifically to the hepatitis B and human papillomavirus (HPV) vaccines [5]. Regarding the HPV vaccine coverage, although the expected target set in the 2014–2019 cancer plan was 60%, [6] the rate of 17-year-old girls having received a complete vaccination schedule was of about 30% in 2013 [7] and decreased to 24% in 2016 to reach up to 30.7% in 2020 [8]. HPV infection is the most common sexually transmitted infection, with more than 80% of sexually active men and women infected by HPV at the age of 45 [9, 10]. Although most people do not develop complications, HPV infection can lead to harmful health consequences, including condylomas, precancerous or cancerous genital lesions (cervix, anal margin, penis) or oropharyngeal cancers [11].

General practitioners (GPs) play a crucial role by ensuring adherence to immunization as a source of health information and providing advice for adolescents and their parents [12–16]. In France, HPV vaccination is recommended from 11 to 14 years old with the possibility of catching-up to 19 years old [17]. As there is no school based immunization programs in France, general practitioners are the main vaccinators. However, vaccine hesitancy affects part of this profession [18, 19]. In a national panel of 1,712 French GPs, more than a quarter did not recommend HPV vaccination to adolescent girls or their parents in 2014 [19]. The barriers to this proposal for HPV vaccination among GPs are relatively well known. They include the fear of AEs, a lack of information on the consequences of HPV infection, the cost of the vaccine, parental reluctance, difficulties in addressing sexuality issues and the forgetfulness of the vaccine proposal [20–22].

These studies do not indicate how GPs discuss HPV vaccination with their patients and parents, and how they deal with their potential vaccine hesitancy. These points are of particular interest as part of a national research program (called “PrevHPV”) aiming at co-developing, implementing and assessing a multicomponent intervention to improve the acceptability, and thus the vaccine coverage, of HPV vaccination in the target population (11-19-year-old girls) [23]. This study is part of the first “diagnostic” phase of the PrevHPV program, and the data obtained will be used to co-develop an intervention aimed at training GPs.

The aim of this study was to explore the positioning and approach toward HPV vaccination in GPs’ routine practice and how GPs manage the reluctance of patients and their families towards this vaccination.

## Methods

### Design

We performed a qualitative study through individual semi-structured interviews based on the grounded theory [24, 25]. Grounded theory is a systematic research methodology that involves the iterative process of data collection and analysis to develop a comprehensive understanding of a phenomenon. This approach allows for the emergence of themes and concepts directly from the data, without preconceived notions, facilitating the development of a theory grounded in the participants’ experiences.

To ensure methodological rigor, we followed the Consolidated Criteria for Reporting Qualitative Research (COREQ) checklist. The COREQ checklist is a set of 32 items designed to enhance the comprehensive reporting of qualitative studies, covering aspects such as study design (theoretical framework, participant selection, setting and data collection), analysis and finding, and the research team’s reflexivity [26].

### Participants and enrolment

A balanced sample was sought by soliciting different networks of physicians known to the researchers to maximize variation in practices, positions, and experiences. Different regions were targeted during the enrolment in order to diversify the practices, the characteristics of the physicians’ patients (income level, socio-cultural background, migration status, etc.) and to take into account disparities in terms of vaccine hesitancy across France [27]. Thus, a purposive sample was obtained based on GPs’ age, gender, practice type (alone or grouped practice, mono or multidisciplinary), location and number of years in practice.

HPV vaccination was not mentioned during the first phone contact to avoid prior research on this topic, or even a change in practices. To compensate the potential loss of activity due to the time spent participating in the research, €175 were given to each GP. The enrolment was stopped when the new data no longer provided new information leading to properties of the categories (data saturation) [28].

### Data collection

The study was conducted in 26 GPs between December 2019 and June 2020 in four French regions. Interviews lasted between 40 and 75 min. Most interviews were conducted in person. The last eight interviews were conducted remotely due to the Covid-19 pandemic.

The interviews were conducted by an experienced investigator (VS), researcher in sociology of health, who presented himself as such to avoid GPs from feeling judged by a peer with regard to their knowledge and practices. This allowed GPs to provide explanations that were probably similar to those they have to provide to their patients and their families when proposing HPV vaccination.

Three researchers (VS, AT, HP) created the interview guide (Supplementary Table 1) based on a literature review to identify the main themes to be addressed [1, 5, 13, 14, 18, 19]. The interview guide was pilot tested and improved as the interviews progressed. The interviewer invited the GPs to talk about their choice of general medicine, their daily practice, the place given to public health issues and how they perceived the physician-patient relationship. They were then asked to discuss their behavior towards vaccination, their opinion on the obligation to vaccinate, and their potential hesitancy. Finally, HPV vaccination was specifically addressed. GPs were asked to explain how they offered this vaccine to patients and their parents and how they managed hesitancy and refusals. Throughout the interview, the GPs were encouraged to use situations they had encountered to express their point of view. At the end of the interview, socio-demographic data were collected to establish their profile.

### Data analysis

An inductive analysis was performed, inspired by the grounded theory approach [24, 25, 29]. This method consisted in making properties and categories obtained inductively, i.e., without formulating *a priori* hypotheses, but by constructing them throughout data collection while adopting a reflective approach of deconstruction-reconstruction that could possibly lead to changes in the interview guide to test new hypotheses.

Each interview was first analyzed individually at the time of data collection. The data collected for each GP were then compared to create new categories based on the observed recurrences.

All interviews were conducted, recorded, and then transcribed in full by the same researcher. The analysis involved several members of the research group: the sociologist and two general practitioners who are teachers and researchers at the university (HP, AT).

## Results

Twenty-six GPs were interviewed, 15 were women. The age of the GPs varied widely, ranging from 29 to 66 years. The number of years in practice also demonstrated a broad spectrum, with practitioners having just started their careers to others with as many as 40 years of experience, indicating a range of expertise levels. The types of practices were diverse, including both solo and grouped practices, with some practitioners working in monodisciplinary setups and others in multidisciplinary environments. The participating GPs were spread across different geographic regions The GPs’ characteristics are presented in Table 1.

**Table 1:** Characteristics of the participating GPs

No.	Gender	Age (years)	Number of years in practice	Type of practice	Department
1	M	66	40	Alone	Seine-St-Denis
2	F	39	12	Grouped, monodisciplinary	Seine-St-Denis
3	F	36	6	Grouped, multidisciplinary	Paris
4	F	50	19	Alone	Paris
5	F	40	11	Grouped, multidisciplinary	Yvelines
6	F	38	4	Grouped, multidisciplinary	Seine-et-Marne
7	F	33	2	Grouped, monodisciplinary	Loire
8	F	43	11	Grouped, monodisciplinary	Loire
9	M	29	1	Grouped, monodisciplinary	Loire
10	F	31	3	Grouped, monodisciplinary	Loire
11	F	46	18	Grouped, monodisciplinary	Loire
12	F	36	8	Grouped, monodisciplinary	Val-d’Oise
13	F	31	0	Grouped, multidisciplinary	Val-d’Oise
14	F	58	29	Grouped, multidisciplinary	Seine-et-Marne
15	M	59	30	Grouped, monodisciplinary	Seine-et-Marne
16	M	37	2	Community Health Centers^a^	Hauts-de-Seine
17	M	35	1	Grouped, multidisciplinary	Val-d’Oise
18	M	36	4	Community health centers	Hauts-de-Seine
19	M	34	3	Health Care Center^b^	Hérault
20	F	33	3	Health Care Center	Hérault
21	F	53	16	Alone	Bouches-du-Rhône
22	M	38	5	Grouped, monodisciplinary	Bouches-du-Rhône
23	M	37	2	Community health centers	Paris
24	M	65	35	Grouped, monodisciplinary	Bouches-du-Rhône
25	F	43	10	Grouped, monodisciplinary	Hauts-de-Seine
26	M	52	23	Grouped, monodisciplinary	Pyrénées-Orientales

M: male, F: female

### Recent measures taken by the French health authorities facilitate vaccination proposal

The lowering of the vaccination age to the 11–14 years age group (2012) [30], its reimbursement by the health insurance (2007) and the extension of the vaccination to boys (2020) [31] were perceived as facilitators by all the GPs interviewed.

According to some physicians, lowering the age of vaccination will avoid the difficult discussion about sexuality in the presence of the parents.

Now, it is much easier for us to say that the age of vaccination is between 11 and 14 years old, or from 14 years old because before that, the topic was really underlying sexual relationships.” (I5F, 40)

Opening vaccination to boys could be considered a necessity to ensure the protection of the entire population. This encouraged previously reluctant physicians to learn new scientific data about vaccination.

I think this will really force me to refresh my knowledge [the rationale for HPV vaccination] because I think that talking about covering an entire population will completely change the benefit-risk ratio.” (I2F, 39)

For some GPs, such a measure, beyond its scientific interest, will contribute to gender equality by ensuring that HPV prevention is not solely the responsibility of women. This could improve their commitment to promote HPV vaccination.

Everyone must be vaccinated. I would not understand the opposite logic as everyone is a vector… The logic where only women who will experience the consequences, should be vaccinated, and protect themselves. No, for me, it is better to protect everyone.” (I18M, 35)

### Organizational barriers: adolescence, missed appointments and missed opportunities to vaccinate

Unlike the childhood period, consultations during adolescence are spaced and relatively unpredictable, making opportunities to address the topic of this non-mandatory vaccination rarer. Since 2–3 doses must be scheduled, this results in a large number of missed appointments.

I said it many times before, this is a population that we do not see very often, because from the age of 7–8 years, we no longer see adolescents. […] We see them again episodically later, sometimes around the age 15–16 years.” (I26M, 52)

### Parental vaccine hesitancy and concerns about sexuality

Parents refusing vaccination for their children because of the risks is related to an intuitive and irrational perception of serious AEs of vaccination “in general” [32]. More specifically for the HPV vaccine, the “lack of hindsight” and thus concerns about the “safety of the vaccine” were particularly reported.

And the parental refusals are due to the lack of hindsight regarding the post-marketing experience, to the fact that the vaccine is not 100% effective, to the fact that it remains a product to be inoculated without really knowing…” (I13F, 31)

This hesitancy goes beyond fears about the perceived risks and seems to be related to the symbolic evocation (verbalized or not) of the first sexual intercourse associated with this vaccine. This seems to be very premature for families at the time the vaccination is proposed.

The refusals that I get come from the parents. […] “She is too young, she is not going to have sex now.” (I3F, 36)

The problem is no longer just about the sexual act itself, but rather who they might have sex with, the potential number of partners they might have, or the possibility that their daughters might have sex with “*someone*”. Parents believe that the HPV vaccine is likely to lead to sexual disinhibition. According to the interviewed GPs, this suspected link with sexual disinhibition could be particularly felt by families as it conflicts with the image they wish to give.

Some people tell me: “My child is not like that, she is not a slut.” (I22M, 38)

If this perception reported by the physicians is correct, there seems to be a double omission by the parents: first, HPV infection circulates widely in the population and does not seem to affect any particular populations [9]; second, they think they can strictly and completely control their children’s choice with respect to their first partner, which is far from being the norm [33].

According to GPs, these reservations about adolescent sexuality are mainly expressed by families for which religion is central. Parents think that they can keep control of their children’s sexuality by instilling in them a certain number of moral values that they must respect (the existence of a single sexual partner throughout life, the absence of sexual intercourse before marriage). For these families, the vaccine would not be necessary because of the virtuous behavior of their children.

However, by presenting sexuality as a taboo, a concept shared by all religious families, these GPs contribute to reinforcing this taboo and to essentializing these categories of population. This could lead to a systematic refusal by these families, even before they have actually proposed the vaccination, and they therefore do not propose it.

Oh yes, the religious reason. They say: “My daughter will never have sex, that’s obvious!” Or “it will be her only partner, she will only have one partner, because meetings are rare at church.” (I15M, 58)

### Heterogeneous methods of approach: easy for some, difficult for others

To convince patients, most GPs rely on scientific data. The “lack of hindsight” on the vaccine is thus put into perspective, and the vaccination coverage of other countries is emphasized. As for sexuality, an analogy can be made with the hepatitis B vaccine. However, some GPs reported not being familiar with the latest data on vaccination and therefore having difficulty overcoming the reluctance of some patients.

#### Emphasizing the responsibility of families: promoting prevention

GPs emphasize the responsibility of families: the fact of mentioning the word “cancer” has a powerful symbolic meaning, likely to influence the decision of families, in particular by involving them in a prevention logic.

- And then, when the parents maintain their position, I explain to them that it is a pity, because cancer is still a leading cause of mortality. (I12F, 36)

#### Using their personal experience and the strength of the relationship built over time

Some physicians may use their personal experience, such as their daughter’s vaccination, when patients ask them about it.

They ask me: “What about you, would you vaccinate your daughter?” And if I say yes, that immediately becomes an argument. (I5F, 40)

The trust built over time is thus taken into account and has a beneficial effect [34, 35]. This was expected since trust is an essential element already pointed out in vaccine hesitancy [1, 36]. However, they must have confidence in their ability to change their patients’ attitudes and behaviours [37].

### Typology of GPs according to their behavior regarding the HPV vaccine proposal

We identified three typologies of GPs according to their proposal strategy for HPV vaccination and their perceived effectiveness in convincing patients to get vaccinated: effective physicians, convinced but unpersuasive physicians, and reluctant physicians.

#### Effective physicians

Effective physicians were characterized by the fact of systematically offering vaccination whenever they had the opportunity (quadrivalent vaccine dTacp-IPV at the age of 11–13 in France, menstruation or contraception, sports certificate, etc.), generally from the age of 11. Most of these GPs reported having no problem with the sexual health approach, but when they feel that patients (or their family) are reluctant, they may also desexualize the vaccine, by taking advantage of the younger vaccination age and focusing on the oncogenic process associated with the virus.

These GPs were mainly women, seeing a socially advantaged or mixed patient population. This proximity in terms of gender and social situation could facilitate the proposal of vaccines and their acceptance by the patients. These physicians thus easily talk about sexual health with their patients.

When faced with the reluctance of families, these physicians address the topic again during subsequent consultations, relying on the temporality of brief interventions such as those used for smoking cessation. Repeating the proposal over time is more likely to be accepted as it accompanies the construction of sexualized bodies during adolescence.

- Ah I think I talk about it all the time actually. It is like with cigarettes or alcohol […] Vaccination is the same, if people tell me “no, it is irrelevant”. I tell them, “Do you have any questions?” I will try to answer them, and then I will raise it again, but gently. (I11F, 46)

Not all patients accept it during the first visit. Often, they say, “We will think about it,” and then I add a note in the medical record: “Talk about Gardasil during the next visit” and when I see them again, I talk about it again. (I17M, 35)

These physicians provide information by adopting a “deliberative” approach [38], discussing with patients, educating them so as to convince them that vaccination is the right thing to do.

#### Convinced but unpersuasive physicians

Convinced but unpersuasive physicians also systematically propose HPV vaccination but do not vaccinate much in practice. GPs in this category see mainly socially disadvantaged or mixed patients.

They attribute the low effectiveness of their proposal, that is not followed by a vaccine injection, to the particularly important reservations of their patients and families about HPV vaccination and to the poor attendance of adolescents in their practice. This reservation seems to be more pronounced when it concerns socially disadvantaged families who rarely consult for preventive reasons but rather in urgent situations [39].

This perceived ineffectiveness can sometimes lead to a drop in vaccination proposals, despite the GPs’ conviction of its usefulness, especially when they feel that the time spent trying to convince people cannot be used for other purposes.

Some of these physicians try to address sexuality-related issues, but find it difficult (e.g., when parents think their daughters are too young to talk about it). Others have difficulties talking about the benefit/risk balance or see that patients do not come back to get the vaccine.

Once they suggest getting a patient vaccinated, they do not systematically propose it again during subsequent consultations, due to the non-mandatory nature of this vaccine in France.

These GPs present themselves as experts whose medical knowledge guides the families, who ultimately remain the decision-makers. They are engaged in an “informative” relationship with their patients [38], who make their own choices based on the information received.

#### Reluctant physicians

Physicians in this category do not systematically propose the HPV vaccination.

First, they are less convinced of the benefit/risk ratio of this vaccine. Most of them expressed doubts after reading articles on the HPV vaccine published in a French journal a few years ago [40, 41]that minimized its interest by reporting an uncertain effect on cervical cancer prevention.

So (she thinks), maybe because I was bred on the review “Prescrire”, I always tend to be suspicious of new things, because new things are not as good as they are supposed to be, especially when there is so much money involved (I2F, 39)

Second, the fear of no longer performing pap smears allowing the early detection of precancerous lesions, was very often reported by these GPs:

In fact, I have a problem with Gardasil, I am a little afraid that people will feel protected, and will thus no longer consult for follow-up pap smears thereafter. (I16M, 37)

Despite their expressed reluctance, they do not discourage patients from getting vaccinated when they specifically request it.

I have mixed feelings about Gardasil, I do not offer it to my patients. I know that my collaborator talks about this vaccine and offers it. If I was alone, I would probably inform my patients more. If they want to get vaccinated, I do it (I2F, 39)

Other strategies may be used: one physician anticipated the degree of adherence to vaccination and offered it only to patients who were likely to accept it. He did not propose it to families for whom he felt religion was important, anticipating a refusal.

However, as these physicians readily explained it, patients and their parents rarely express a desire to be vaccinated, given the lack of information available to the general public on this subject. Patients requesting to be vaccinated against HPV are only people with a certain level of income, and they often have a high level of education. This passive attitude could thus contribute to increase social inequalities in health.

They can sometimes become more actively involved and offer the HPV vaccine to high-risk patients. The proposal is then based on the interpretation of various information collected on the patient’s sexuality and the protection methods used, including condoms. This may be problematic because there is evidence that using condoms is unlikely to be sufficient to prevent HPV infection [42].

## Discussion

### Summary

This qualitative study highlighted some difficulties related to the HPV vaccine proposal among GPs. In addition to a low consultation rate of adolescents leading to incomplete vaccination schemes, parental vaccine hesitancy remained a challenge. The factors contributing to parental vaccine hesitancy included the perception that the risks outweigh the benefits and the fact that the HPV vaccine is associated with the onset of sexual activity. The lowering of the target age, the reimbursement of the vaccine by the health insurance and the extension of the target population to boys were considered facilitating institutional factors by the GPs interviewed.

Our study identified three typologies of GPs with different behaviors towards the HPV vaccine proposal: effective physicians, convinced but unpersuasive physicians, and reluctant physicians. Ultimately, the acceptance of the vaccination and its effective implementation often seemed to depend on the level of persuasion of the physician regarding the benefits of this vaccine, [18, 19] on the repetition of the proposal over time and on the management of the symbolism of entry into sexuality associated with this vaccine.

### Comparison with the existing literature

Many of the barriers identified in our study support those already identified in the literature: the fear of AEs, the lack of information on the consequences of HPV infection, parental reluctance and difficulties in addressing sexuality issues [20, 21, 43].

A French qualitative study has been conducted in 2016 among GPs, gynecologists and pediatricians and aimed to understand the decision-making processes leading physicians to recommend this vaccine. The results of this study in terms of physician classification were similar to ours, in particular with regard to the use of informative and deliberative approaches [44].

The impact of the GPs’ persuasion level regarding the benefits of this vaccine on vaccination acceptance and its effective implementation has also been reported in a cross-sectional observational study conducted in 2014 in a national panel of 1,712 randomly selected GPs in private practices in France [19].

### Implication for research and practice

The low medical attendance of adolescents suggests the need for a systematic proposal of vaccination to optimize the rare contacts with this population [45]. An evidence-based medical decision support tool incorporating recent data confirming the impact of vaccination on reducing cervical cancer [46–48] could influence the behavior and practice of reluctant physicians who do not systematically propose the HPV vaccine [49].

Providing in an easy-to-remember format information on the benefits, risks, and data from other countries on the beneficial impact of a high vaccine coverage could allow physicians to overcome their own resistance and that of patients and their parents. This update of knowledge, including the currently available evidence that the HPV vaccine reduces cervical cancer risk, [46–48] could reduce or even overcome the vaccine hesitancy of some GPs who reported doubts about vaccination [50, 51].

Patient-centered communication techniques such as brief interventions or motivational interviewing could then be used to improve vaccine adherence, especially for convinced but unpersuasive physicians. As the nature of the reported vaccine hesitancy differed according to the socio-cultural characteristics of the patient populations, communication attitudes and techniques will have to be adapted to them. Desexualization of the vaccine could be particularly useful for physicians who have difficulty discussing sexuality with their youngest patients or who consider this topic taboo because of supposed cultural standards. The apprehension of some physicians to suggest the HPV vaccination, related to the socio-cultural characteristics of the families, was associated with the risk of anticipating a systematic refusal by some families, even before they actually offered it.

All these elements will be taken into account to guide the next phase of this project, both in the development of a tool to assist with making a decision to be vaccinated and the creation of a training program for physicians adapted to HPV vaccination.

Other elements that could contribute to increasing vaccination coverage (catch-up campaigns, promotion of sexual education campaigns for adolescents and implementation of vaccination counseling with other healthcare workers involved in adolescents’ health) were not specifically addressed in this study, but may have been part of other prevHPV project studies in the diagnostic, co-construction and intervention phases [52–54].

### Strengths and limitations

The number of interviews conducted and the diversity of the profiles of the physicians interviewed in terms of demographics and practices allowed achieving data saturation and identifying the various behaviors of physicians toward HPV vaccination. Based on our findings and the highly pragmatic dimension of our study, the analysis of these different behaviors of physicians towards vaccination will allow us to offer them an evidence-based medical decision support tool as well as a specific training in motivational interviewing techniques [55] allowing them to adapt their behavior and practice based on what works while avoiding what does not.

Being interviewed by a non-medical person and knowing it, the GPs were able to discuss more freely about the vaccination and sometimes used pedagogy, by simulating how they would present the vaccine to young girls who would be candidates for vaccination.

Although our study focuses on doctors, and therefore does not question the patients themselves about their willingness to accept HPV vaccination, some of the comments reported by GPs illustrate certain components of the 5/7 C model [56, 57]. For example, the extension of vaccination to boys, which goes hand in hand with the prospect of protecting everyone, is linked to collective responsibility. Doctors’ arguments are aimed at providing information that will help patients improve their perception of the vaccine’s benefit-risk balance (calculation and complacency), as well as reinforcing their trust in HPV vaccination and in the rightfulness of this public health initiative (confidence).

This study also has several limitations. Physicians were encouraged to estimate whether more or less than one in two people would be willing to be vaccinated. However, this arbitrary figure was relatively intuitive for physicians, while being close to the vaccination coverage rate of 60% recommended in the cancer plan [6].

None of the participating GPs reported to be overtly anti-vaccine, contrary to what has been described in the literature [43]. However, only 3% of French GPs are highly hesitant or opposed to vaccination [58]. This limitation should be considered minor because the purpose of this study was to obtain information allowing us to create a tool that could be used by GPs likely to offer this vaccination.

The phenomenon of vaccine hesitancy is a complex and dynamic issue that is influenced by a variety of individual and contextual factors. Given the COVID-19 pandemic and the unprecedented vaccine campaigns, it is possible that specific factors influencing vaccine hesitancy have emerged since this study was conducted [59]. Therefore, the findings should be interpreted within the specific context and time period in which they were collected.

In France, general practitioners are the ones who initiate HPV vaccination the most [12], however, the target population of 11–14 year-olds also consults gynecologists and pediatricians: their opinions on the subject are also of interest, but were not specifically addressed in this study.

The first 18 interviews were conducted in-person, while the subsequent 8 interviews were conducted remotely. This variation in the mode of interview delivery could potentially introduce methodological bias. In-person interviews may elicit different nuances and non-verbal cues (gestures, eye contact, …) compared to remote interviews [60]. It is crucial to acknowledge that this difference in interview settings might have influenced the depth and richness of the data collected. While we strived to maintain consistency in interview questions and facilitation techniques, the variation in interview settings should be considered as a potential limitation in the interpretation of our findings.

### Electronic supplementary material

Below is the link to the electronic supplementary material.

Supplementary Table 1: interview guide

## Data Availability

The data supporting the results of this study are available on request from first author A.T., but restrictions apply to the availability of these data. The interview transcripts, even if pseudonymized, may provide elements of identification which, in view of European regulations on the protection of personal data, prompt the authors not to make these data public. The data is, however, available from the authors on reasonable request and with the authorization of the PrevHPV consortium.
