# Supplementary material for: Barriers and facilitators to the HPV vaccine: a multicenter qualitative study of French general practitioners
Source: Arch Public Health. 2024 Jan 4;82:2. doi: 10.1186/s13690-023-01227-8 (PMC10768163; doi:10.1186/s13690-023-01227-8)
Supplement: Supplementary file 1 — Supplementary Table 1: interview guide [file 13690_2023_1227_MOESM1_ESM.docx]

**Supplementary Table 1: interview guide**

**Introduction**:

- Before starting the interview, begin with a brief presentation of the topic of the survey (a qualitative survey on vaccination practices in general practice, with a particular focus on HPV vaccination)
- Remind participants of the interview procedure (open-ended questions; estimated duration: about 1 hour and 15 minutes; anonymity and confidentiality of the data collected; consent to interview recording)
- Then, collect oral consent.

**Each theme begins with an opening question that should allow the interviewees to express themselves freely. The questions proposed in each theme of the grid should be asked if any information is missing.**

**1] The GP: his professional career path.**

**Opening question:** To begin with, can you introduce yourself and tell me about the nature of your activity here.

- Why did you choose general practice? / What is your idea of being a general practitioner?
- What type of patients do you see?
  - Do you see more young or older patients? Do you rather see patients belonging to the wealthy class or in a precarious situation? What types of illnesses are you dealing with?
- What is the size of your patient population?

**2] The practices of the general practitioner**

**Opening question:** We will start by talking about your medical practices. Generally speaking, how would you describe your relationships with your patients?

- What place do you give to informing patients during a consultation?
- What part does the patient play in decision making? Do you ever make decisions for them? Do you ever completely follow their point of view? (e.g., cardiovascular risks, antibiotic therapy)
- What is the place of prevention, vaccination and more generally public health issues in your practice?
- In general, what place do you give to sexual health during your consultations? How often do you talk about it?
- Are there any patients with whom you do not feel comfortable?
- How do you deal with the issue of adverse drug reactions with your patients?

**3] Vaccination practices of the general practitioner**

**Opening question:** We will now talk about vaccines, in general. Are vaccines an issue for you? (or: Do you have any reservations about vaccination in general?)

- What do you think about vaccines, whether recommended or mandatory, for example, MMR [measles, mumps, rubella] vaccines, Neisvac [meningitis], HBV vaccines?
- What do you think about mandatory vaccination?
- Do you have any reservations for some vaccines? For what reasons? (benefits/risks)
- Do you start by providing information about these vaccines? Do you use specific tools to inform your patients? [flyers, posters, etc.].
- For infants, do you always talk about vaccines or are there situations that are more appropriate than others (if so, which ones?)? Do you sometimes forget certain vaccines?
- For adolescents, do you sometimes forget vaccines? In which situations? And for adults?
- Are there any patients to whom you do not suggest getting vaccinated? For what reasons? Do you have any examples?
- For what reasons may your patients be reluctant when you talk to them about getting vaccinated? (If necessary, give examples: adjuvants, multiplicity of vaccines, etc.) What is your own opinion?
- How do you react when patients seem reluctant? What about refusals?
- How do you get information about vaccines? / What are your sources of information?

**4] HPV vaccination: Knowledge of guidelines**

**Opening question:** We will now talk more specifically about the HPV vaccine. To begin, what do you know about HPV vaccines?

- What do you know about their efficacy, their risks, their benefits for individual patients, their usefulness for population health?
- In your practice, did you experience situations involving high-grade lesions or cervical cancer? How did it influence your practice?
- In your practice, did you experience situations of serious adverse events related to the HPV vaccine? How did it influence your practice?
- Do you have any reservations about these vaccines?
- Do you feel insufficiently trained or informed about this vaccination? What are your training needs?
- Do you feel comfortable with the guidelines for HPV vaccination?
- In your opinion, what place do you give to the prevention of cervical cancer using Pap smears?
- Do you have any concerns about the involvement of the pharmaceutical industry in promoting this vaccine?

**5] HPV vaccination: Practices**

**Opening question:** Now let’s turn to your practices. How do you talk about HPV vaccination? Do you find it difficult?

- When you offer HPV vaccination, do you talk about the diseases that will be prevented? (cervical cancer, condylomas, precancerous lesions, etc.)
- Is there an age from which you feel more comfortable offering the vaccine? In connection with your answer, do you offer it to younger people? to older people?
- In what situations do you sometimes not advise this vaccination to your patients? What could prevent you from offering this vaccination? (lack of time, inappropriate situation, etc.)
- A public consultation is ongoing regarding the extension of HPV vaccination to boys. Will you offer HPV vaccination to boys? Do you already offer it? In what situations?
- Apart from situations where the vaccine was not indicated, have you ever advised against HPV vaccination? For what reasons?
- In the context of HPV vaccination, do you discuss sexuality issues with your young patients, alone or with their parents? Do you find it difficult?
- Do you think that discussing this vaccination with the parents and not directly with the young girls might be a barrier for you? Why or why not?

**6] GPs facing vaccine hesitancy in the context of HPV**

**Opening question:** What are the most common patient reluctances you face? What do you think about it?

- When you offer this vaccination, do you think it is rather well accepted? Do you vaccinate more or less than any other general practitioner?
- In the last month, did you experience any vaccination refusal? Can you tell me more about it? Do you plan to discuss it again with this patient during the next consultation?
- How do you manage patient refusals?
- What arguments do you use?
- Do you talk about the potential risks of HPV vaccines? How do you formulate this risk approach?

**7] Socio-demographic characteristics and practice patterns**

- What year were you born?
- What was your father's occupation or industry?
- How long have you been practicing?
- What is your practice sector? [sector 1, sector 2]
- Do you see patients without an appointment? Do you make home visits?
- What is your practice setting? [individual, monodisciplinary group, multidisciplinary group]
- Do you have any other medical activities? [Teaching, research, etc.]
- Have you taken any other health care training? If yes, which ones?
- Are you affiliated with a union? If yes, which one?
- Do you practice alternative medicine? [mesotherapy, naturopathy, etc.]

Are you a member of a professional organization? If yes, which one?
